# MicroRNA-149 is downregulated in Alzheimer’s disease and inhibits β-amyloid accumulation and ameliorates neuronal viability through targeting BACE1

**DOI:** 10.1590/1415-4757-GMB-2020-0064

**Published:** 2021-01-11

**Authors:** Wenyan Du, Chengbin Lei, Yong Dong

**Affiliations:** 1 Zibo Central Hospital, Department of Science and Education, Zibo, Shandong, China.; 2 Zibo Central Hospital, Department of Clinical Laboratory, Zibo, Shandong, China.; 3 The Affiliated Hospital of Qingdao University, Department of Blood Transfusion, Qingdao, Shandong, China.

**Keywords:** MicroRNA-149, Alzheimer’s disease, BACE1, diagnosis, APP

## Abstract

Beta-site amyloid precursor protein cleaving enzyme 1 (BACE1) plays a critical role in Alzheimer’s disease (AD) pathogenesis. This study aimed to investigate the relationship between microRNA-149 (miR-149) and BACE1, and evaluate the clinical significance and biological function of miR-149 in AD progression. Bioinformatics analysis and a luciferase reporter assay were used to confirm the interaction between miR-149 and BACE1. Expression of miR-149 and BACE1 was estimated using quantitative real-time PCR. The clinical significance of miR-149 in AD diagnosis and severity determination was evaluated using ROC analysis. The effect of miR-149 on Aβ accumulation and neuronal viability was analyzed in Aβ-treated SH-SY5Y cells. miR-149 was found directly binding the 3’-UTR of BACE1 and was negatively correlated with BACE1 in AD patients and cell model. Serum miR-149 expression was downregulated in AD patients and served as a potential diagnostic biomarker. The overexpression of miR-149 in Aβ-treated SH-SY5Y cells resulted in inhibited Aβ accumulation and enhanced neuronal viability. This study demonstrated that serum miR-149 is decreased in AD patients and serves as a candidate diagnostic biomarker, and that the overexpression of miR-149 may suppress Aβ accumulation and promote neuronal viability by targeting BACE1 in AD model cells.

## Introduction

Neurodegenerative diseases, encompassing Alzheimer disease (AD), Parkinson’s disease (PD), amyotrophic lateral sclerosis (ALS), multiple system atrophy (MSA), Huntington disease (HD) and frontotemporal dementia (FTD), make up a series of pathologies characterized by different etiologies with distinct features in morphology and pathophysiology (Sheikh *et al*., 2013). AD is one the most frequent neurodegenerative diseases and represents a leading threat to human health in aging population (Atkinson 2017). The pathogenesis of AD involved the formation of β-amyloid (Aβ) plaques, neuronal cell viability and microglia activation (Zhang and Zheng 2019). Thus, numerous studies focus on AD therapeutic strategies regarding to the regulation of these important pathological events (Chen 2018). Currently, the therapeutic strategies for AD had no abilities to prevent or delay disease development, leading to the urgent need to further understand AD pathogenesis, which may provide novel molecular therapeutic targets (Gao *et al*., 2016). Aβ accumulation represents a central event in AD progression, which is produced by the cleavage of amyloid precursor protein (APP) by β-secretases, such as beta-site amyloid precursor protein cleaving enzyme 1 (BACE1) (Panza *et al*., 2019). BACE1 has been widely investigated in AD and contributes to the development and progression of AD (Das and Yan, 2019). Thus, researchers have focused on the inhibitors of BACE1 to improve the treatment of AD (Panza *et al*., 2019).

An increasing number of studies have reported that BACE1 could be directly inhibited by microRNAs (miRNAs) in AD progression (An *et al*., 2017; Ji *et al*., 2019). miRNAs are a group of small non-coding RNAs with important biological function in the progression of various human diseases (Mohr and Mott, 2015; Rupaimoole and Slack, 2017). They can regulate gene expression by directly binding to the 3’-untranslated region (3’-UTR) of the target messenger RNA (mRNA) (Zhang *et al*., 2018). A variety of aberrantly expressed miRNAs have been found in the progression of neurodegenerative diseases with attractive clinical significance and important biological function (Quinlan *et al*., 2017). Some miRNAs have been identified as a regulator of BACE1 and thus serve as potential therapeutic targets in AD (Wang *et al*., 2008; Li and Wang, 2018). In this study, a putative binding site of microRNA-149 (miR-149) was found at the 3’-UTR of BACE1. The decreased expression of miR-149 has been reported in ALS, which is another kind of neurodegenerative disease (Dardiotis *et al*., 2018). In addition, the regulatory effect of miR-149 on blood-brain barrier and neuronal cell proliferation has been previously reported (Xu *et al*., 2017; Wan *et al*., 2018). However, there is little published data on the expression and biological function of miR-149 in AD.

The critical role of BACE1 in combination with the putative binding site of miR-149 at BACE1 prompted us to explore the role of miR-149 in AD. In this study, we attempt to confirm the relationship between miR-149 and BACE1 and evaluate the expression and clinical significance of miR-149 in AD patients. In addition, the effect of miR-149 on Aβ accumulation and neuronal viability was assessed in an AD cell model, which was constructed by Aβ treatment in SH-SY5Y cells. This is the first study to understand the role of miR-149 in AD, and the findings may make a contribution to the diagnosis and treatment of AD.

## Material and Methods

### Patients and serum collection

The experimental protocols of this study were approved by the Ethics Committee of Zibo Central Hospital. A total of 112 AD patients, were recruited from Zibo Central Hospital between April 2014 and December 2017. The diagnosis of AD was performed with the diagnosis criteria of National Institute of Neurological and Communication Disorders and Stroke/Alzheimer’s disease and Related Disorder Association (NINCDS-ADRDA) (McKhann *et al*., 1984). In addition, 60 healthy volunteers were enrolled from the individuals who received physical examination in Zibo Central Hospital. There was no statistical difference in age, gender and education duration between the AD patients and healthy controls. Blood samples were collected and centrifuged to prepare serum samples. Among the 112 AD patients, 16 patients provided cerebrospinal fluid (CF) samples. To confirm the expression changes of miR-149 in other neurodegenerative diseases, serum samples were collected from 18 PD patients. All the collected samples were stored at -80 ℃ for further use. To evaluate the dementia severity of AD patients, the Mini-Mental State Examination (MMSE) score was recorded with following definition: 21 ≤ MMSE score ≤ 26 represents mild dementia; 15 ≤ MMSE score ≤ 20 represents moderate dementia; MMSE score < 15 represents severe dementia (Kahle-Wrobleski *et al*., 2017). Each participant provided a written informed consent.

### Cell culture and treatment

A human neuroblastoma cell line SH-SY5Y and a human renal epithelial cell line HEK293 were purchased from the Cell Bank of Type Culture Collection of Chinese Academy of Sciences (Shanghai, China) and cultured in Dulbecco’s modified Eagle’s medium (DMEM; Invitrogen, Thermo Fisher Scientific, CA, USA) in a humidified incubator with 5% CO_2_ at 37 ℃. For the construction of AD cell model, SH-SY5Y cells were harvested and treated with 1 µM of Aβ25-35 (Sigma-Aldrich, Saint Louis, MO, USA) for 24 h to simulate AD progression. 

### Cell transfection

The expression of miR-149 in SH-SY5Y cells was evaluted by cell transfection, which was performed by Lipofectamine 3000 reagent (Invitrogen, Carlsbad, CA, USA) according to the manufacturer’s instruction. The miR-149 mimic (50 nM; GenePharma, Shanghai, China) was used to upregulate the expression of miR-149 *in vitro*, and the mimic negative control (50 nM; mimic NC; GenePharma, Shanghai, China) sequences were used as controls. Cells were subjected to Aβ treatment at 24 h after cell transfection.

### RNA extraction and quantitative real-time PCR (qRT-PCR)

Total RNA in serum, CF and cells was extracted by TRIzol reagent (Invitrogen, Carlsbad, CA, USA), and cDNA was synthesized from RNA using a PrimeScript RT reagent Kit (Takara, Dalian, China) following the manufacturer’s instruction. To evaluate the relative expression of miR-149 and mRNA expression of BACE1, qRT-PCR was performed by a SYBR Green I Master Mix kit (Invitrogen) and the 7500 Real-Time PCR System (Applied Biosystems, USA). The final expression values were calculated by the 2^−ΔΔCt^ method, and U6 and GAPDH were respectively used as internal controls for miR-149 and BACE1.

### Luciferase reporter assay

To analyze the interaction between miR-149 and BACE1, a luciferase reporter assay was used. The wild type (BACE1 3’-UTR WT) or mutant type (BACE1 3’-UTR MT) of BACE1 3’-UTR was cloned into pGL3-luciferase basic vector (Promega, Madison, WI, USA). miR-149 mimic or mimic NC was co-transfected into HEK293 cells with the reporter vector by Lipofectamine 3000 reagent (Invitrogen) based on the manufacturer’s instruction. At 24 h post-transfection, the relative luciferase activity was measured by the Luciferase 1000 Assay System (Promega, USA). 

### Western blot assay

After cell lysis by RIPA buffer (Beyotime, Shanghai, China), the proteins in SH-SY5Y cells were quantified by a BCA protein assay kit (Beyotime). The proteins were first separated by SDS-PAGE and transferred to polyvinylidene fluoride (PVDF) membranes (Millipore, Billerica, MS, USA). After 2 h of blocking with 5% nonfat milk, the membranes were incubated with the primary antibody anti-APP overnight at 4 ℃ followed by HRP-conjugated secondary antibody at room temperature for 1 h. At last, the membranes were visualized by an enhanced chemiluminescent substrate (BioRad, USA).

### MTT assay

A 3-(4,5-dimethylthiazol-2-yl)-2,5-dimethyl tetrazolium bromide (MTT) cell proliferation assay kit (Sigma-Aldrich, MO, USA) was used to evaluate SH-SY5Y cell viability. SH-SY5Y cells with a density of 5 × 10^3^ cell/well were seeded into 96-well plates and incubated at 37 ℃ for 3 days. At the time points of 0, 24, 48 and 72 h, 0.5 mg/mL MTT was added into the wells for 4 h at 37 ℃. Then, the MTT solution was removed and 100 µL dimethyl sulfoxide (DMSO) was added into the wells. The cell viability was examined by reading the absorbance at 490 nm using a microplate reader (BioRad, California, USA).

### Statistical analysis

All statistical analyses were carried out by SPSS 18.0 software (SPSS Inc., Chicago, IL) and GraphPad Prism 5.0 software (GraphPad Software, Inc., USA). Data were expressed as mean ± SD, and each experiment was repeated at least 3 times. Differences between groups were analyzed using Student’s *t*-test or one-way ANOVA followed by Tukey’s test. Correlation between indicators was analyzed using the Pearson correlation coefficient. A receiver operating characteristic (ROC) curve was plotted based on serum miR-149 expression, and the area under the curve (AUC) was computed. A *P* < 0.05 indicated statistically significant.

## Results

### BACE1 serves as a direct target gene of miR-149

**Figure 1 -:**
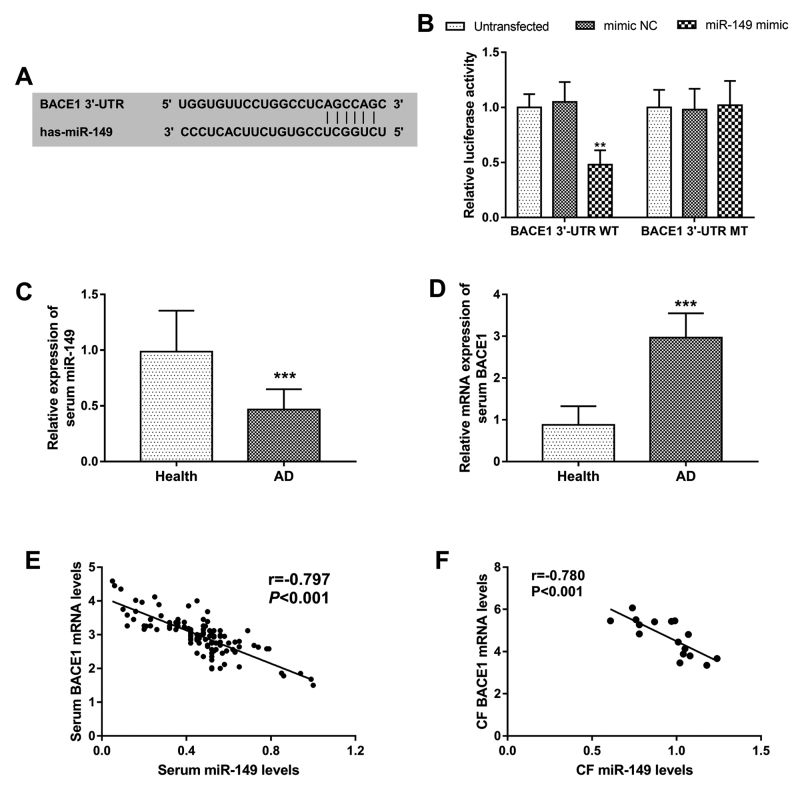
miR-149 directly binds the 3’-UTR of BACE1. **A**. The putative binding site of miR-149 at the 3’-UTR of BACE1. **B**. The luciferase activity results to confirm the interaction between miR-149 and BACE1. **C**. Serum expression of miR-149 detected by qRT-PCR. **D**. Serum mRNA expression of BACE1 measured by qRT-PCR. **E**. A negative correlation between serum miR-149 and BACE1 (r = -0.797, *P* < 0.001). **F**. A negative correlation between CF miR-149 and BACE1 (r = -0.780, *P* < 0.001). CF: cerebrospinal fluid; **P < 0.01; ***P < 0.001.

The prediction result obtained from miRanda (http://www.microrna.org/microrna/home.do) showed a putative binding site of miR-149 at the 3’-UTR of BACE1 (Figure 1A). A subsequent luciferase reporter assay was performed to verify the interaction of miR-149 with BACE1. As shown in Figure 1B, the relative luciferase activity in BACE1 3’-UTR WT group was significantly inhibited by the overexpression of miR-149 (*P* < 0.01), while no change was observed in the BACE1 3’-UTR WT group at the relative luciferase activity (*P* > 0.05). These results suggested that BACE1 was a direct target gene of miR-149.

### Expression of miR-149 in AD patients and is negatively correlated with BACE1

Considering the interaction of miR-149 and BACE1, the expression of miR-149 and BACE1 in AD patients was estimated by qRT-PCR. The results shown in Figure 1C and 1D revealed that the serum expression of miR-149 was decreased in AD patients, whereas the mRNA of BACE1 was increased in AD patients compared with the healthy controls (both *P* < 0.001). Furthermore, serum miR-149 levels were found to be negatively correlated with the serum mRNA levels of BACE1 in AD patients (r = -0.797, *P* < 0.001, Figure 1E). In addition, the expression of miR-149 and BACE1 in CF samples from 16 AD patients was evaluated, and a negative correlation between CF miR-149 and BACE1 was also found in AD patients (r = -0.780, *P* < 0.001, Figure 1F).

### Relationship of miR-149 with dementia severity in AD patients 

**Figure 2 -:**
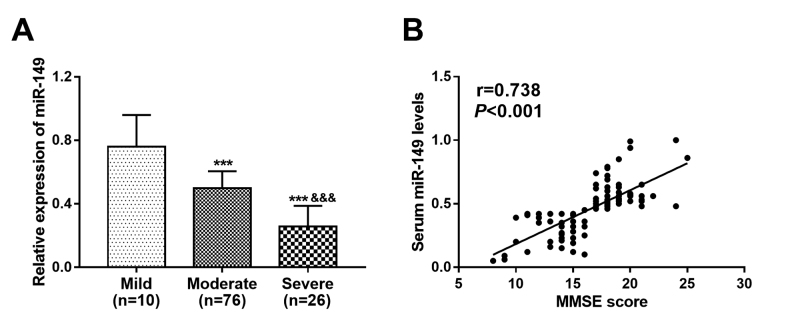
Expression of miR-149 in AD patients with different degree of dementia and its correlation with MMSE scores of AD patients. **A**. Expression of miR-149 was decreased as disease severity increased. **B**. A positive correlation between miR-149 and MMSE score (r = 0.738, *P* < 0.001). ***P < 0.001 vs. Mild; ^&&&^ *P* < 0.001 vs. Moderate.

MMSE score is an indicator to reflect the cognitive function of AD, and the patients in this study were classified into mild dementia (n = 10), moderate dementia (n = 76) and severe dementia (n = 26) groups according MMSE score. The lowest expression of miR-149 was found in patients with severe dementia, and the highest miR-149 expression was observed in patients with mild dementia (all *P* < 0.01, Figure 2A). In addition, a positive correlation was found between the serum miR-149 levels and the MMSE scores of AD patients (r = 0.738, *P* < 0.001, Figure 2B)

### Clinical significance of serum miR-149 in patients with AD

**Figure 3 -:**
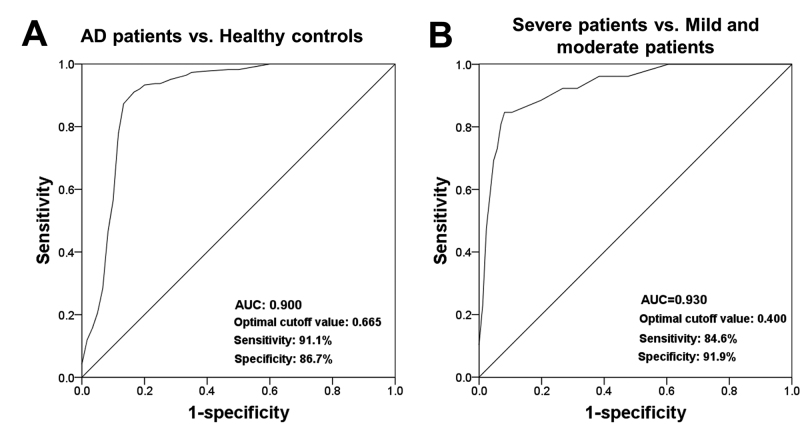
ROC curves based on serum miR-149 levels in AD patients. **A**. A ROC curve based on serum miR-149 to distinguish AD patients from healthy volunteers. **B**. A ROC curve based on serum miR-149 to screen AD cases with severe dementia from AD patients with mild and moderate dementia. AUC, area under the curve.

This study evaluated the diagnostic value of miR-149 for the differentiation between AD patients and healthy controls by a ROC analysis. The ROC curve shown in Figure 3A exhibited an AUC of 0.900 for the serum miR-149 expression levels in AD patients with a sensitivity of 91.1% and a specificity of 86.7% at a cutoff value of 0.665, suggesting the relatively high diagnostic value of serum miR-149. Furthermore, miR-149a levels in AD patients with different degree of dementia were used to plot anther ROC curve (Figure 3B). The AUC value was 0.930 and the optimal cutoff value was 0.400 with a sensitivity of 84.6% and a specificity of 91.9%, indicating that serum miR-145 might be an indicator to predict the severity of AD.

### Differential expression of serum miR-149 between AD patients and PD patients

**Figure 4 -:**
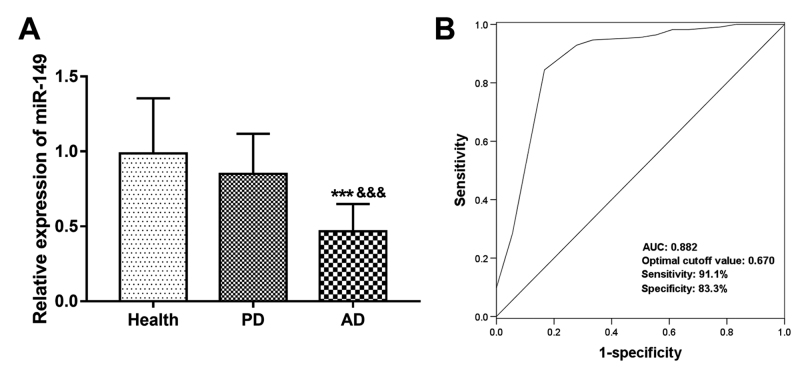
Differential expression of serum miR-149 between AD and PD patients. A. Serum miR-149 was significantly lower in AD patients than that in PD patients. B. A ROC curve based serum miR-149 expression to distinguish AD cases from PD cases. AUC, area under the curve.

To confirm the specific role of miR-149 in AD among the neurodegenerative diseases, the expression of serum miR-149 in PD patients was assessed. As shown in Figure 4A, serum miR-149 expression had no statistical difference between healthy controls and PD patients (*P* > 0.05), but was significantly downregulated in AD patients compared with PD patients (*P* < 0.01). Furthermore, the ROC curve based on serum miR-149 levels in AD and PD patients showed that miR-149 could be used to distinguish AD cases from PA patients with an AUC of 0.882, sensitivity of 91.1% and specificity of 83.3% (Figure 4B).

### Expression of miR-149 in AD cell model and its regulatory effect on BACE1 expression

**Figure 5 -:**
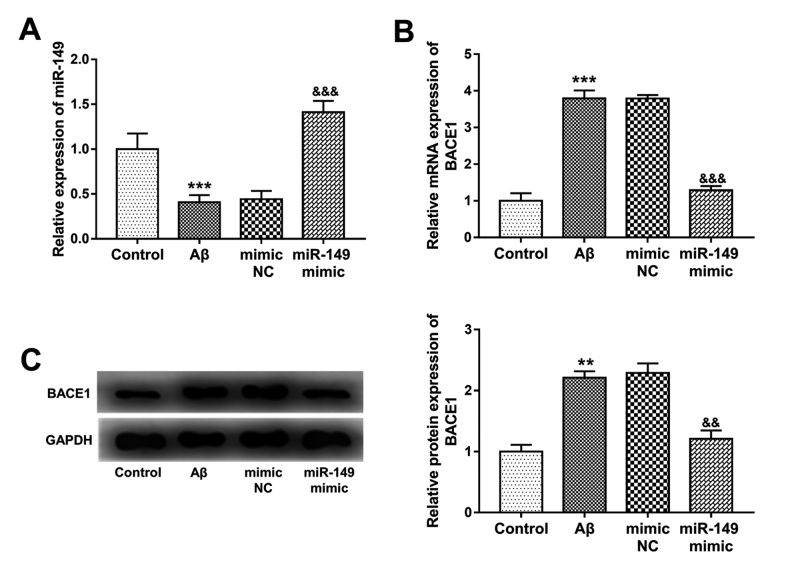
Expression of miR-149 in Aβ-treated SH-SY5Y cells and its negative regulatory effect on BACE1 expression. **A**. Aβ treatment in SH-SY5Y led to inhibited miR-149 expression, but cell transfection with miR-149 mimic significantly promoted the expression miR-149. **B**. The increased mRNA expression of BACE1 in Aβ-treated cells was inhibited by the overexpression of miR-149. **C**. The protein expression of BACE1 in Aβ-treated cells with miR-149 overexpression. **P < 0.01, ***P < 0.001 vs. Control; ^&&^ *P* < 0.01, ^&&&^ *P* < 0.001 vs. Aβ.

Consistent with the expression results of miR-149 in AD patients, the expression of miR-149 in AD cells, that were constructed by Aβ treatment, were significantly reduced (*P* < 0.001, Figure 5A). By cell transfection, the inhibited miR-149 expression in AD cells were upregulated by miR-149 mimic (*P* < 0.001). As expected, both the mRNA and protein expression of BACE1 in Aβ-treated SH-SY5Y cells were significantly upregulated (*P* < 0.001, Figure 5B and 5C). In the AD model cells with overexpression of miR-149, the Aβ-induced BACE1 upregulation was inhibited by miR-149 (*P* < 0.001), which confirmed the negatively regulatory effect of miR-149 on BACE1 in AD progression.

### Effect of miR-149 on Aβ accumulation and neuronal viability

**Figure 6 -:**
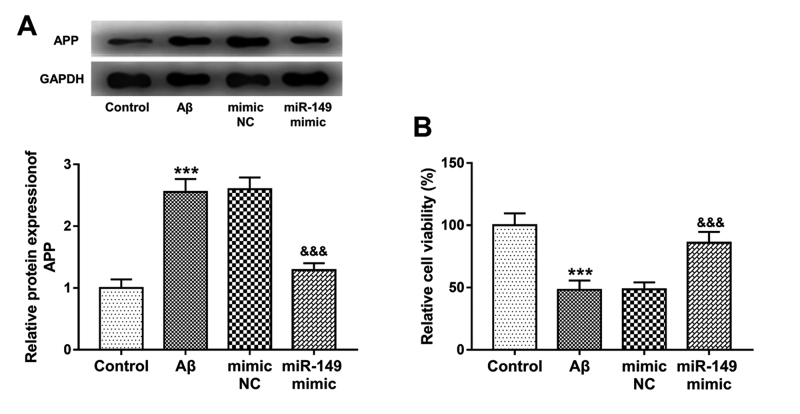
Effect of miR-149 on Aβ accumulation and neuronal viability in Aβ-treated SH-SY5Y cells. **A**. The increased APP protein expression by Aβ treatment was reversed by the overexpression of miR-149. **B**. The decreased neuronal viability induced by Aβ was promoted by the upregulation of miR-149. ***P < 0.001 vs. Control; ^&&&^ *P* < 0.001 vs. Aβ.

In the Aβ-treated SH-SY5Y cells, Aβ accumulation was promoted that indicated by the increased protein levels of APP (*P* < 0.001, Figure 6A). Additionally, the cell viability of SH-SY5Y cells was significantly inhibited by Aβ treatment (*P* < 0.001, Figure 6B). In the cells with miR-149 overexpression, the regulatory effects of Aβ treatment on APP expression and cell viability were both abolished, which evidenced by the decreased APP protein expression and increased cell viability (all *P* < 0.001).

## Discussion

Several studies have highlighted the important role of miRNAs in various human diseases (Qiu *et al*., 2015). There are also some functional miRNAs that serve pivotal roles in the pathogenesis of AD. Hou *et al*. (2020) found that miR-124 was aberrantly expressed in AD and targeting the miR-124/PTPN1 signaling was determined as a potential therapeutic strategy of AD. Wu *et al*. (2020) demonstrated the increased expression of miR-592 in AD rat model and provided evidence for miR-592 to inhibit neuronal cell viability. Ji *et al*. (2019) performed a study to investigate the role of miR-22-3p in AD progression, which showed that miR-22-3p overexpression could inhibit Aβ deposit by targeting MAPK14. These studies suggested that further understanding about functional miRNAs in AD progression may provide novel therapeutic targets for AD therapy.

BACE1 plays a critical role in the pathogenesis of AD, which promotes Aβ accumulation by enhancing the cleavage of APP (Koelsch, 2017). As a result, the methods to inhibit BACE1 has attracted increasing attention for their therapeutic potential in AD treatment (Yan and Vassar, 2014). Of note, some miRNAs, that could directly inhibit BACE1, have been proposed as candidate therapeutic targets in AD. For instance, the decreased miR-340 in AD has been found to regulate the accumulation of Aβ and neuronal survival by targeting BACE1 (Tan *et al*., 2020). Another study also focused on the inhibitors of BACE1 and found that miR-298 served as an upstream regulator of BACE1 and was involved in AD development (Chopra *et al*., 2020). In this study, a miR-149 putative binding site at the 3’-UTR of BACE1 was found by bioinformatics analysis, and the subsequent luciferase activity results demonstrated that BACE1 was a direct target of miR-149. In AD patients, the serum expression of miR-149 was found to be reduced when compared to healthy control, which was consistent with the expression results in ALS, which is another kind of neurodegenerative diseases (Dardiotis *et al*., 2018). A negative correlation was obtained between the serum miR-149 levels and serum mRNA expression of BACE1, and the CF miR-149 was also negatively correlated with BACE1 in the CF samples of AD patients, indicating the potential regulatory relationship between miR-149 and BACE1 in AD. Furthermore, in an AD cell model that was constructed by Aβ treatment in SH-SY5Y cells, the overexpression of miR-149 could significantly inhibit the expression of BACE1. These results further demonstrated that miR-149 could directly inhibit BACE1 in AD. An increase in the expression of miR-149 was observed in AD patients with severe dementia, and serum miR-149 levels were positively correlated with MMSE scores of AD patients, which indicated that miR-149 was associated with AD severity. Thus, we suspected that miR-149 might be involved in the progression of AD by directly targeting BACE1.

miRNAs have been determined to be a group of good diagnostic tools in a variety of human diseases, including AD (Swarbrick *et al*., 2019). Serum miR-193a-3p (Cao *et al*., 2020) and miR-133b (Yang *et al*., 2019) have been determined to be candidate biomarkers in AD diagnosis. The diagnostic value of miR-149 has been demonstrated in other diseases, such as human malignancies (Ow *et al*., 2018) and bipolar disorder (Choi *et al*., 2017). Considering the dysregulation of serum miR-149 in AD patients, this study evaluated its clinical significance in AD diagnosis. The ROC analysis results of this study implied that the decreased serum miR-149 had relatively high diagnostic accuracy in the differentiation between AD patients from healthy controls. In addition, the markedly decreased serum miR-149 was found in AD when compared to PD patients, which had diagnostic accuracy to distinguish AD and PD cases. Thus, the decreased serum miR-149 might serve as a promising diagnostic biomarker of AD. By using the serum miR-149 levels in AD patients with different degree of dementia, we found that serum miR-149 could also distinguish severe AD cases from mild and moderate AD patients, indicating that serum miR-149 might be a candidate indicator to predict the severity of AD.

It is well known that BACE1 can contribute to the accumulation of Aβ, which is one of the major characteristics of AD pathogenesis, leading to abnormal nerve signaling, neuroinflammation and impaired neuronal cell viability (Penke *et al*., 2017). In this study, the increased protein expression of APP in Aβ-treated SH-SY5Y cells was significantly reduced by the overexpression of miR-149, indicating that miR-149 overexpression might suppress Aβ accumulation. The regulatory effect of miR-149 on cell viability has been previously reported in neuroblastoma and vascular smooth muscle cells (Mao *et al*., 2019, Zhang *et al*., 2019). In this study, the cell viability of Aβ-treated SH-SY5Y cells was significantly inhibited, while this inhibition was abolished by the upregulation of miR-149, indicating the neuroprotective role of miR-149 in AD progression.

In conclusion, this study revealed that BACE1 serves as a direct target of miR-149, and that miR-149 can decrease Aβ accumulation and improve neuronal viability in AD cell model by targeting BACE1. In addition, serum decreased miR-149 may be a candidate diagnostic biomarker and a potential indicator for disease severity in AD patients. These findings may provide evidence for a novel diagnostic biomarker and a potential therapeutic target for AD therapy.
